# Grazing Rates of *Calanus finmarchicus* on *Thalassiosira weissflogii* Cultured under Different Levels of Ultraviolet Radiation

**DOI:** 10.1371/journal.pone.0026333

**Published:** 2011-10-18

**Authors:** David M. Fields, Caroline M. F. Durif, Reidun M. Bjelland, Steven D. Shema, Anne B. Skiftesvik, Howard I. Browman

**Affiliations:** 1 Bigelow Laboratory for Ocean Sciences, West Boothbay Harbor, Maine, United States of America; 2 Institute of Marine Research, Austevoll Research Station, Storebø, Norway; University of Hamburg, Germany

## Abstract

UVB alters photosynthetic rate, fatty acid profiles and morphological characteristics of phytoplankton. Copepods, important grazers of primary production, select algal cells based upon their size, morphological traits, nutritional status, and motility. We investigated the grazing rates of the copepod *Calanus finmarchicus* on the diatom *Thalassiosira weissflogii* cultured under 3 levels of ultraviolet radiation (UVR): photosynthetically active radiation (PAR) only (4 kJ-m^−2^/day), and PAR supplemented with UVR radiation at two intensities (24 kJ-m^−2^/day and 48 kJ-m^−2^/day). There was no significant difference in grazing rates between the PAR only treatment and the lower UVR treatment. However, grazing rates were significantly (∼66%) higher for copepods feeding on cells treated with the higher level of UVR. These results suggest that a short-term increase in UVR exposure results in a significant increase in the grazing rate of copepods and, thereby, potentially alters the flow rate of organic matter through this component of the ecosystem.

## Introduction

Reduction in stratospheric ozone is linked to increases in ultraviolet radiation (280–400 nm), e.g. [1], [2], and damaging UVB (280–320 nm) levels. While ozone layer depletion and concomitant increases in UVB are greatest over the poles, pronounced increases at mid-latitude areas of the Northern and Southern Hemispheres have also been reported (e.g. [1]). During the Norwegian spring and summer, significant levels of UVB are present as early as 05:00 h, and as late as 22:30 h (Browman, unpublished data) and can penetrate water to considerable depths [3], [4], [5]. Extended daily exposures, superimposed upon increases related to ozone depletion, likely induce UVB damage to susceptible aquatic organisms.

Oceanic primary productivity accounts for 40–50% of global carbon fixation [6]. Ultraviolet radiation, even at its current level, is harmful to aquatic organisms and reduces the net productivity of many marine ecosystems (e.g. [5], [7],[8],[9]). UVB can have a range of inhibitory effects on algae (see [10]), including changes in morphology and nutrient uptake [11], [12], damage to DNA and to light transduction and carbon assimilation mechanisms [13], [14], [15], as well as alterations in fatty acid composition and other nutritional components of cells [16], [17].

The indirect effects of UVB damage are often compounded through the ecosystem causing broad-scale changes in trophic interactions [18] and in the biogeochemical cycling of key organic and inorganic components. While it is well known that UVR exposure has damaging effects on primary producers (directly), surprisingly little is known about its indirect effects, for example on the grazing rates of mesozooplankton feeding on UV-exposed algae and, thereby, its potential effects on the transfer rate of organic matter through the food chain. Metazoan grazers such as copepods are significant consumers of primary production and provide an important food source for higher trophic levels, from larval fish to whales [19]. Unassimilated phytoplankton cells pass through the guts of copepods and are packaged into rapidly sinking fecal pellets that contribute to the vertical flux of organic matter out of the euphotic zone [20]. Given the potential importance of mesozooplankton for trophic energy transfer and export efficiency, it is essential that the effects of environmental factors on the grazing rates of copepods are accurately parameterized.

Zooplankton feeding on algae cultured under high doses of UVB radiation generally under-perform in terms of growth and egg production rates [21]. However, it remains unclear if these effects are a product of the quality of the food or if they are the result of a decrease in ingestion rate. Experiments from different algae-grazer combinations, primarily from freshwater systems, have produced inconsistent results, with some reporting increased ingestion rates [22] while others report a decrease [23]. This study adds to this limited data base by investigating the grazing rates of *Calanus finmarchicus* adults on the marine diatom *Thalassiosira weissflogii* cultured under tightly controlled and carefully characterized levels of UVR.

## Materials and Methods

### Study species

Diatoms and copepods were selected for this study because they are important components of the planktonic communities of many temperate marine environments, including the North Atlantic. Specifically, *Calanus finmarchicus* constitutes up to 70% of the mesozooplankton biomass over a wide area of the northeast Atlantic during summer [24] and is, as such, an important species. *Calanus finmarchicus* adults underlie the wasp-waist trophic structure for several whale species [25] and their nauplii are food for fish larvae [26].

*Thalassiosira weissflogii* is a common coastal diatom species with a long history of use in laboratory grazing rate experiments e.g. [27], [28], [29]. In the laboratory, *T. weissflogii* responds to moderate levels of sustained UVR exposure by producing protective compounds, including mycosporine-like amino acids (MAAs) (e.g. [30]).

### Algal cultures

*Thalassiosira weissflogii* (CCMP #1052) were cultured at a constant 15∶9 light∶dark photoperiod in autoclaved seawater enriched with filtered and sterilized F/2 medium (Guillard). Algae were reared at 19 (±1.5) °C in 3 replicate 1.5 L quartz flasks. Cultures were grown until they reached the stationary phase (after 161 hours). Once the maximum concentration was indentified, cultures were maintained in exponential growth phase using semi-continuous batch cultures to keep cell counts at 40–70% maximum carrying capacity of the treatment. To maximize cultures' surface area exposure to UV, cultures were transferred to10 L Teflon® bags (Welch Fluorocarbon, Dover, NH) with four replicates for each light treatment. Growth rates were calculated as the log of the change in concentration over time. Counts of algal cells were made using a Beckman Coulter Z2 Coulter Counter.

### Spectral treatments

There were three spectral exposure treatments: UV-depleted (PAR-only, Treatment 1), Ambient-UV (Treatment 2) and enhanced-UV (Treatment 3) produced by using, respectively, (1) 4 GE lamps (General Electric Polylux X2 F36W/830), (2) 4 GE and 1 UV Q-panel lamps (Q-Lab UVA-340; Q-Lab, Cleveland, OH), and (3) 4 GE and 2 Q-panel lamps. All lamps were aged for 100 hours before the experiment began. Algae received a total of 193 hours (∼8 days) of exposure prior to being fed to the copepods.

Spectral irradiance was measured using an OL-754-O-PMT (Gooch and Housego, Orlando, Florida, USA) spectroradiometer. The integrating sphere (100 mm diameter) was placed inside the culture bags in water. Measurements were also taken in the air with the sphere placed outside of the bags to obtain values for transmission through the bags. In both sets of measurements, the edge of the sphere was positioned 14 cm from the lamps. Irradiance values for measurements taken in water inside the culture bags were corrected using an immersion correction factor (ICF) for each wavelength to account for changes in optical properties when measurements are made in water. The ICFs used here are those derived for this probe by the manufacturer [31].

**Figure 1:**
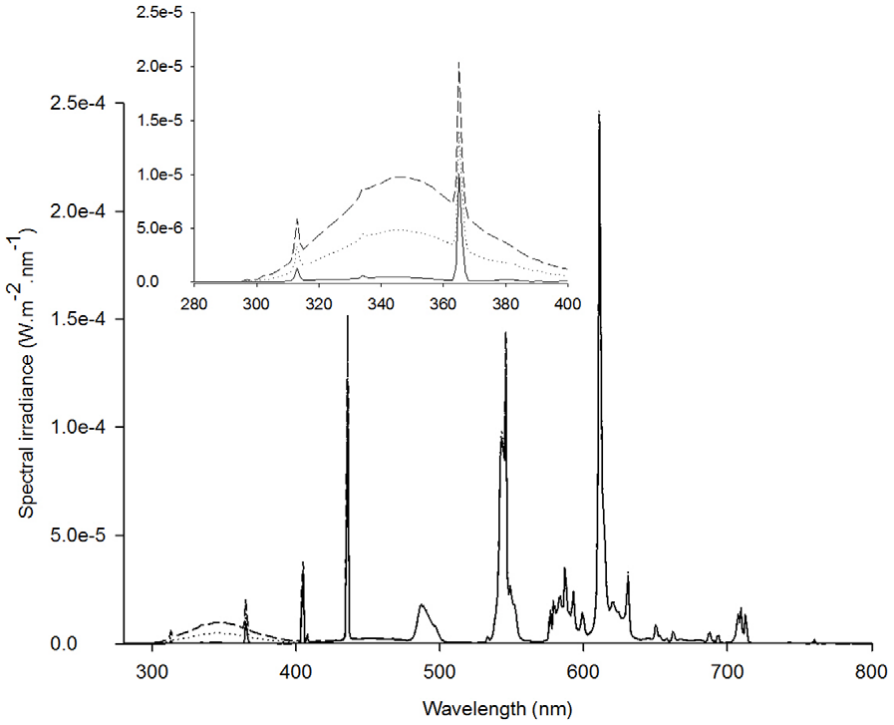
Spectral irradiance for the 3 treatments: PAR (solid line), PAR plus ambient UVR (dotted line), and PAR plus enhanced UVR (dashed line) measured inside the Teflon bags that were used for culturing the algae.

Irradiance spectra are presented in Figure 1 and daily irradiances in the UVB (280–320), UVA (320–400 nm), and photosynthetically active radiation (PAR; 400–700 nm) per treatment in Table 1. Ambient radiation data, collected by the Norwegian UV monitoring network, was obtained from the Norwegian Radiation Protection Authority (NRPA). NRPA uses a multi-channel radiometer (305, 313, 320, 340 and 380 nm; GUV-541, Biospherical Instruments, CA) situated in Bergen (60°22′43″N, 5°20′33″E, University of Bergen), 22 km north of Austevoll, where algae and copepods were cultured. Average daily UVB irradiance measured in Bergen between June 1^st^ and July 31^st^ in 2008 and 2009 was approximately 40 kJ m^−2^.

**Table 1:** Daily irradiance of UVB, UVA and PAR provided to *Thalassiosira weissflogii* cultures used as food for *Calanus finmarchicus*.

		Daily irradiance (kJ m^−2^)		
		Waveband UVB	Waveband UVA	PAR
Treatment		(280–320 nm)	(320–400 nm)	(400–800 nm)
PAR	in air	6	51	4108
	in bag	4	36	2974
UVR	in air	33	347	4250
	in bag	24	260	3156
UVR+	in air	72	762	4567
	in bag	48	512	3113

Algal cultures received one of three treatments: PAR-only (PAR), PAR plus ambient UVR levels and PAR plus enhanced UVR (UVR+).

The depth to which UV penetrates water is highly variable [3]. In a Norwegian fjord, penetration was mostly dependent on the concentration of CDOM, colored dissolved organic matter [32]. In most systems, downwelling irradiance at 305 nm decreased by at least one order of magnitude within the first meter below the surface [32]. The ambient-UV treatment (Treatment 2) used in this study (24 kJ m^−2^ UVB), although lower than the daily average in air, was ecologically relevant relative to what occurs in the surface layer of the water column during a Norwegian summer. The enhanced-UV treatment (Treatment 3) represented a 2-fold increase in UVB and UVA compared to the ambient-UV treatment (Treatment 2). PAR was equivalent in all treatments.

### Copepod grazing

Copepods (*Calanus finmarchicus*) were reared in large 5000 L flow-through silos on a mixed diet of *Rhodomonas baltica*, and *Isochrysis sp.* at food levels of 2*10^4^ cells mL^−1^ at the Institute of Marine Research's Austevoll Research Station, Norway. Individual adult stage copepods were handpicked from the culture and placed into 2L Erlenmeyer flasks. There were 9 control and 9 treatment flasks (3 replicates per light treatment in both control and experimental flasks), each treatment flask contained 15 individuals while control flasks contained only the algae at 2*10^4^ cells mL^−1^. In the treatment flasks, the copepods were fed algae that had previously been exposed to either 1) PAR-only 2) ambient-UV or 3) enhanced-UV radiation for 8 days. Because copepods often show abnormally high feeding rates during the first several hours of a grazing experiment [27], [33], [34], experimental flasks were allowed to acclimate for 24 hours prior to measuring grazing rates. Experiments were run for 48 h in the dark at 15°C. All experimental vessels and controls were gently bubbled to maintain algae in suspension.

Counts of algal cells were made using a Beckman Coulter Z2 Coulter Counter. Ingestion rates were calculated from cell counts of all the controls and each beaker containing grazers based on the equations developed by Frost [27].

### Statistical analyses

Two-way ANOVA was used to compare growth rates of algae with spectral treatments and replicate flasks as factors. One-way ANOVA was used to compare average algal cell diameters between spectral treatments and average ingestion rates of copepods between treatments. Pairwise multiple comparisons were used (Holm-Sidak method, α = 5%) for both ANOVAs to determine which treatments were significantly different.

## Results

### Algal culture

Although all the algal cultures received the same dose rate of PAR, the treatments showed very different growth rates and reached significantly different maximum concentrations (Table 2). Growth rates of algae were normally distributed (Shapiro-Wilk normality test, p = 0.15) and variances were homogeneous (Bartlett test, p = 0.86). PAR-only treated cells (Treatment 1) showed the highest growth rates and enhanced-UV treated cells (Treatment 3) grew approximately one order of magnitude slower. Differences were significant between PAR-only (Treatment 1) and the two other treatments, ambient-UV and enhanced-UV (ANOVA p<0.001). There was no significant difference between replicates within each treatment (ANOVA, p = 0.252). The maximum concentration reached in the PAR-only treatment (1) was approximately 16% greater than the ambient-UV treatment (2) and nearly 500% more than the enhanced-UV treatment (3) (Table 2).

**Table 2:** Characteristics of *Thalassiosira weissflogii* cultures grown in F/2 media under 3 light conditions.

	*PAR*	*UV*	*UV+*
Cell Size (µm)	12.2 (1.4)	12.9 (1.8)	12.7 (1.7)
Cell Volume (µm^3^)	950.1 (1.4)	1124.0 (3.1)	1072.5 (2.6)
Growth Rate (day^−1^)	0.74 (0.24)	0.28 (0.26)	0.08 (0.26)
Maximum Concentration (cell mL^−1^)	1.4×10^5^	1.2×10^5^	2.6×10^4^

See Table 1 for spectral information. One standard deviation is given in parentheses.

Average cell diameters were significantly different between all spectral treatments (ANOVA, F = 267.458, df = 2, p<0.001). Ambient-UV treated cells (2) were the largest followed by enhanced-UV (3) treated algae (1.1% smaller) and then PAR-only treated (Treatment 1) algae (5.7% smaller).

### Ingestion rate

**Figure 2:**
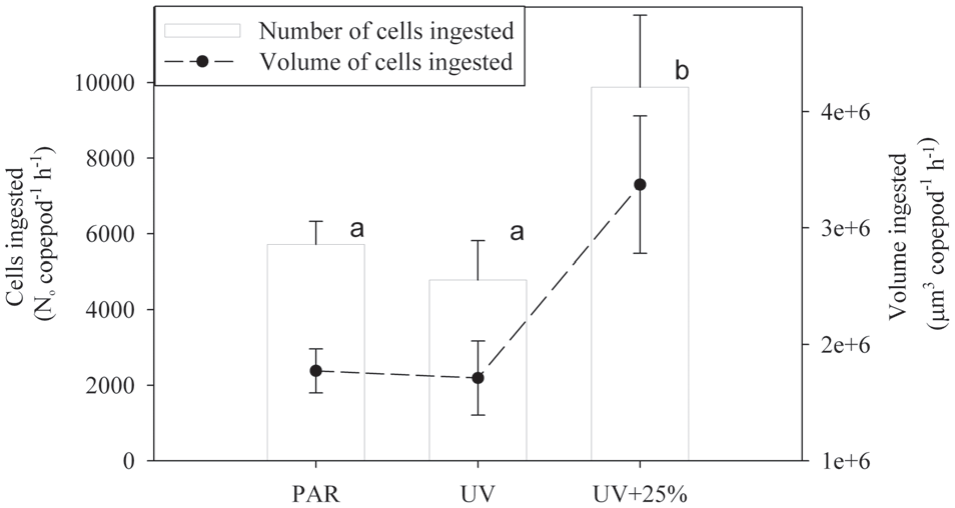
The number of cells (left axis) and volume of cells ingested by *Calanus finmarchicus* feeding on *Thalassiosira weissflogii*. Algae were cultured under one of 3 different light treatments: PAR only (PAR; Treatment 1), PAR plus ambient UVR (UV; Treatment 2), or PAR plus enhanced UVR (UV+; Treatment 3). Grazing rates were measured over a 48 h feeding cycle. Ingestion rates were significantly higher in the UV+ treatment. No significant difference was found in the grazing rates between the PAR- and ambient-UV-treated cells. Lower case letters indicate homogeneous groups after the ANOVA and post-hoc test (see methods for details).

Ingestion rates in all experiments were normally distributed (Shapiro-Wilk normality test, p = 0.96) and variances were homogeneous (Bartlett test, p = 0.22). There were significant differences in the grazing rates of *Calanus finmarchicus* feeding on the algae grown under the different light treatments (One way ANOVA - F = 8.724, df = 2, p = 0.017). Ingestion rate of enhanced-UV treated algae (Treatment 3) was significantly different from those of PAR-only and ambient-UV treated algae (Fig. 2). However, there was no significant difference in the ingestion rates of *C. finmarchicus* grazing on cells grown under PAR-only and ambient-UV (Treatments 1 and 2).

## Discussion

Grazing rates of copepods feeding on laboratory-reared algae are used to estimate egg production, trophic transfer rates and to parameterize models of food web structure. However, most of our laboratory-based knowledge of the grazing rates of copepods is based on algae cultured under PAR, devoid of any UVR. In this study, we measured the grazing rates of *Calanus finmarchicus* feeding on *Thalassiosira weissflogii* cultured under two different ecologically-realistic levels of UVR.

*UVR effects on Thalassiosira weissflogii.* UVR reduces nutrient uptake in algae which leads to physiological changes analogous to those resulting from nutrient limitation [35], [36]. These changes include diminished growth rates, decreased cell counts [37], [38] and changes in cell size (Table 2). Although the general deleterious effects of UVR on phytoplankton are well known [5], [7], [8], [39], not all species are equally susceptible to UV damage. Some phytoplankton species are UVR tolerant as a result of protective pigmentation [30], [40], increase in cell size [17], [23], changes in morphology [41] or more efficient repair mechanisms [42]. *T. weissflogii* produces UVR protective pigments, including mycosporine-like amino acids (MAAs), in response to long-term exposure (16–22 days) to UV radiation [30]. Once adapted to moderate levels of exposure, *T. weissflogii* shows no difference in growth rates or photosynthetic capacity supporting suggestions that, when adapted, this diatom is relatively tolerant of UVR [30]. Under oceanic conditions with a well-mixed upper water column, however, organisms rarely receive long-term (weeks) exposure to UV radiation. In contrast to phytoplankton that have been exposed to UVR for long periods, short-term exposures (8 days) caused notable effects on *T. weissflogii*. Consistent with this, we observed a significant decrease in growth rate and maximum culture density (under finite nutrient concentrations), and a significant increase in cell size (Table 2).

*Ingestion of UVR treated cells.* Ingestion rates of *Calanus finmarchicus* were 66% greater on cells exposed to high UVR compared to cells that received PAR only. Kouwenberg and Lantoine [38] found that *C. helgolandicus* produced significantly fewer eggs with lower hatching success when fed UV-exposed cells. Although the number of fecal pellets released were similar between treatments, no direct measurements of algal cell ingestion rates by the copepods were made in those experiments. In combination with our study, however, these results support the hypothesis that decreased growth and egg production rates of zooplankton fed on a diet of UVR-treated cells are the result of decreased nutritional value (quality) rather than decreased ingestion rate (quantity). Although the underlying mechanisms driving the higher ingestion rate cannot be resolved from our experiment, possible explanations may be that the grazing rates are affected by changes in cell morphology (size or shape); [27], [43], [44], [45], [46], or indirect effects such as increased cell fragility or decreased digestibility [23], [47].

Although copepods, including *Calanus* spp., selectively graze on larger cell sizes [27], [48] because they are more readily detected [49], the small differences in size found in this study are unlikely to be responsible for the large increase in ingestion rate. The largest difference in cell size occurred between the PAR-only treatment (Treatment 1) and the ambient-UV (Treatment 2), supporting previous results showing that cells exposed to UVR are larger. However, despite this nearly 6% difference in diameter (18% increase in volume), we found no significant difference in grazing rates between these treatments. A comparison between the UV treatments (Treatment 2 and 3), however, produced only a 1.5% difference in cell diameter with Treatment 2 (ambient- UV) producing larger cells but being grazed at significantly lower rates. If differences in cell size were the main driver of the observed differences in grazing rates we would have expected Treatment 2 to be ingested at the highest rate.

Reproductive success is affected by the quantity and nutritional quality of maternal diets [50]. Nutritional quality has been shown to be as important for successful reproduction as food quantity [51], [52]. The nutritional deficiency hypothesis [53], [54], [55] states that lack of essential compounds in marine copepod diets induces a decrease of egg production, hatching success, and larval survival. Changes in the nutritional quality of phytoplankton cells exposed to high UVR appears to be species-specific and dependant on the proxy used. Arctic diatoms show a significant decrease in growth rate due to UV exposure but relatively little effect on the fatty acid profile [56], [57]. In contrast, temperate diatom species showed significant decrease in nearly all characterized fatty acids and significantly lower total hydrolysable amino acid (THAA) in UV-treated cells than cells exposed to PAR only. The finding that *C. helgolandicus* produced significantly fewer eggs with lower hatching success when fed UV-exposed cells clearly shows that for copepods, UV-treated cells are of lower quality. To meet nutritional needs, copepods grazing on lower quality food would require a proportional increase in quantity of food ingested. For primary grazers such as copepods, nutrient deficient cells are considered poor food for a variety of reasons. One major effect of UVR exposure is considerable thickening of the cell wall due to glycoprotein accumulation [23], [47] which decreases assimilation efficiency in the gut. UVR exposure also modifies the biochemical profiles of cells [16], [58], often resulting in a reduction in amino acids and essential fatty acids [17]. Even low UVB exposures (12 kJ m^−2^ day^−1^) over short durations (4 days) altered the FA profile of a suite of marine diatoms [16]. Goes et al. [16] report significant increases in the saturated (SAFA) and monounsaturated (MUFA) fatty acids and large decreases in the polyunsaturated fatty acids (PUFA). Fatty acid profiles–specifically PUFA levels–play a primary role in animal nutrition and are critical for egg production and hatching success in marine copepods [54]. Results from this study show that when grazing on diatoms exposed to high UVR, copepods increase their feeding rates. When considered together with previous studies showing decreased growth and reproductive rates associated with feeding on UV-treated cells, our results suggest that the increased ingestion rate observed is insufficient to offset the combination of decreased digestibility and lower nutritional value of the UV-treated algae.

Results from this study–showing that *C. finmarchicus* consume a significantly higher number of cells in the high-UV treatment—support the hypothesis that copepods consume more cells when they are of lower nutritional quality. This could have consequences for the efficiency with which organic matter is transferred through the food web.
